# The effectiveness of smoking cessation interventions for socio-economically disadvantaged women: a systematic review and meta-analysis

**DOI:** 10.1186/s13643-022-01922-7

**Published:** 2022-06-02

**Authors:** Nicola O’Connell, Emma Burke, Fiona Dobbie, Nadine Dougall, David Mockler, Catherine Darker, Joanne Vance, Steven Bernstein, Hazel Gilbert, Linda Bauld, Catherine B. Hayes

**Affiliations:** 1 The Discipline of Public Health and Primary Care, The Institute of Population Health, Trinity College Dublin, Russell Centre, Tallaght Cross, Dublin, D24 DH74 Ireland; 2 Usher Institute and SPECTRUM Consortium, University of Edinburgh, Old Medical School, Teviot Place, Edinburgh, EH8 9AG UK; 3 School of Health and Social Care, Edinburgh Napier University, Sighthill Court, Edinburgh, EH11 4BN UK; 4 Trinity College Library, Trinity College Dublin, Dublin 2, Ireland; 5 Irish Cancer Society, 43/45 Northumberland Road, Dublin 4, Ireland; 6 Emergency Medicine, Yale University School of Medicine, 464 Congress Avenue, Suite 260, New Haven, CT 06519-1315 USA; 7 Department of Primary Care and Population Health, University College London Medical School, Royal Free Campus, Rowland Hill Street, London, NW3 2PF UK

**Keywords:** Health disparities, Tobacco control, Behavioural, Cessation, Meta-analysis, Gender

## Abstract

**Introduction:**

This systematic review and meta-analysis assessed the effectiveness of smoking cessation interventions among women smokers in low socio-economic status (SES) groups or women living in disadvantaged areas who are historically underserved by smoking cessation services.

**Methods:**

A systematic literature search was conducted using MEDLINE (OVID), EMBASE, Cochrane, CINAHL, PsychINFO and Web of Science databases. Eligibility criteria included randomised controlled trials of any smoking cessation intervention among women in low SES groups or living in socio-economically disadvantaged areas. A random effects meta-analysis assessed effectiveness of interventions on smoking cessation. Risk of bias was assessed with the Cochrane Risk of Bias tool. The GRADE approach established certainty of evidence.

**Results:**

A total of 396 studies were screened for eligibility and 11 (6153 female participants) were included. Seven studies targeted women-only. 5/11 tested a form of face-to-face support. A pooled effect size was estimated in 10/11 studies. At end of treatment, two-thirds more low SES women who received a smoking cessation intervention were more likely to stop smoking than women in control groups (risk ratio (RR) 1.68, 95% CI 1.36–2.08, *I*^*2*^*=* 34%). The effect was reduced but remained significant when longest available follow-up periods were pooled (RR 1.23, 95% CI 1.04–1.48, *I*^*2*^ = 0%). There was moderate-to-high risk of bias in most studies. Certainty of evidence was low.

**Conclusions:**

Behavioural and behavioural + pharmacotherapy interventions for smoking cessation targeting women in low SES groups or women living in areas of disadvantage were effective in the short term. However, longer follow-up periods indicated reduced effectiveness. Future studies to explore ways to prevent smoking relapse in this population are needed.

**Systematic review registration:**

PROSPERO: CRD42019130160

**Supplementary Information:**

The online version contains supplementary material available at 10.1186/s13643-022-01922-7.

## Background

Tobacco use is one of the most common preventable causes of premature death, killing more than eight million people a year globally [1]. Worldwide, it is estimated nearly one in seven adults smoke tobacco daily [2]. Smoking is a leading cause of cancer linked to increased risk of at least 12 different cancers [3], as well as non-malignant respiratory diseases, cardiovascular disease, reproductive issues, early menopause and many other chronic health conditions [4].

Socio-economic status (SES) refers to the social and economic circumstances that influence how people are positioned within the structure of society [5]. Tobacco use deepens and maintains societal health inequalities [6] and accounts for one third of the differences in life expectancy between lower and higher SES groups [7]. The overall reduction in smoking rates in Europe has been a public health success, but the main effects have been observed in middle- and high-income groups, causing an increase in health inequity [8]. In 2015 and 2016 in the USA, smoking prevalence was 10% for adults in higher income households, compared with 25% in adults living below the US Federal Poverty Level [9]. Research suggests that as disadvantage accumulates within an individual (e.g. experiencing unemployment, poverty, disability and psychological distress), the risk of smoking increases [10]. Low SES smokers also face disproportionate smoking-related illness compared to affluent smokers [8].

Gender inequalities in smoking initiation, cessation and the health consequences of smoking also exist. Inequity in mortality from smoking-related conditions accounts for 6% of the overall inequity in the female death rate in European countries [11]. There are also specific gender differences in adverse health effects. Women smokers experience a higher incidence of myocardial infarction [12]; are more likely to be diagnosed with depression and more often use smoking to control weight or mood than men [13]. Female smokers have additional risk for gender-specific cancers, e.g. breast [14] and cervical cancer [15]. In Europe, while lung cancer rates in men have declined by 9.2% between 2015 and 2020, the rate in women has increased by 6% over the same period. Predicted mortality from lung cancer In women is now higher than that of breast cancer, and the number of deaths from lung cancer is now greater than breast cancer [16]. Women may also be less likely to quit or attempt to quit than men, with UK data showing that while women are more likely to access services (e.g. cessation support) than men, they are less likely to stop smoking [17].

Smoking cessation reduces smoking-related disease and premature death [18]. Stopping smoking at age 30 increases life expectancy by a decade [19]. Nicotine replacement therapy (NRT) is the most widely used smoking cessation pharmacotherapy. All licensed forms of NRT (gum, transdermal patch, nasal spray, inhalator, tablets and lozenges) increase quit rates by 50–60% compared to a placebo or no-NRT control group, regardless of setting [20]. Varenicline (Champix) and bupropion (Zyban) can reduce withdrawal symptoms and relieve cravings, with evidence that combined treatment of both drugs can improve abstinence rates, particularly for highly dependent and heavy smokers [21].

Behavioural interventions for smoking cessation vary in intensity, frequency, mode of contact (e.g. group, telephone and face-to-face support) and provider type. Tobacco cessation behavioural change techniques (BCTs) have been summarised by Michie et al. [22] as those that address: motivation; self-regulatory capacity and skills; adjuvant activities; and other BCTs, e.g. building rapport and relapse prevention. There is strong evidence that providing in person or telephone behavioural support in combination with pharmacotherapy is likely to increase the chance of quitting by between 10–20%, compared to less intensive or no behavioural support [23].

Women from affluent areas are more likely to be successful in their smoking cessation attempts than women from poor areas [24, 25]. Suggested reasons for lower success rates in low SES women are structural causes such as: limited access to effective smoking cessation interventions [26]; higher rates of physical comorbidities; lower income; more stressful living environments; less leisure time; and higher involvement in unpaid caregiving roles. Important psychosocial factors include stigma associated with smoking, fear of being judged; higher levels of stress; fear of failure; potentially less support from family or community in smoking cessation attempts; smoking as a means to help control weight, lack of knowledge about existing services; and perceptions that interventions are ineffective or too expensive [27, 28]. Given these complex factors likely operate in a way that are specific to low SES women compared to affluent women or low SES men, there is a need for interventions that specifically target this group.

Behavioural interventions targeting smoking cessation in the general population may increase health inequalities [29] as they are likely to be more effective in higher than lower SES groups. There have been increasing international calls for interventions to address socio-economic differentials by tailoring interventions to the needs of lower SES smokers. The World Health Organisation (WHO) through the Framework Convention of Tobacco Control has highlighted the need for approaches tailored to gender when developing tobacco control policies in the light of increasing cancer rates, particularly increasing lung cancer in women [30] and they argue for gender-specific risks in tobacco usage to be addressed when developing tobacco control strategies [31]. An evidence-to-practice gap currently exists in developing targeted gender-based approaches. While previous reviews of smoking cessation interventions show promise in improving smoking cessation rates in disadvantaged groups including at-risk youth, individuals with mental illness and low-income smokers [32, 33], only one previous meta-analysis has examined the effectiveness of smoking cessation interventions among low-SES women.

This review examines the existing evidence of effectiveness of smoking cessation interventions tailored to low SES women smokers and women smokers living in disadvantaged areas, the majority of whom are in low SES. The term ‘low-SES women’ denote both groups. The review excludes smoking in pregnancy which is the subject of a separate on-going systematic review [34].

## Methods

### Search strategy and selection criteria

This systematic review followed PRISMA-P guidelines [35]. It assessed programme effectiveness, recruitment strategies, retention, drop-out and follow-up rates, strategies that enhance implementation and barriers and enablers to successful implementation. All comparator groups, as defined by study authors, were included. Only randomised controlled trials (RCTs) were included in our search.

The databases MEDLINE (OVID), EMBASE, Cochrane, CINAHL, PsychINFO and Web of Science were searched (see Supplementary Material 1 for study search strategy). Studies were included if they

(i) included information on women aged 18 years and over who smoke and were socio-economically disadvantaged or lived in an area of socioeconomic disadvantage (“low-SES”);
(ii) reported the results of any smoking cessation intervention, with no limitations on intervention type;
(iii) contained any definition of ‘neighbourhood socioeconomic disadvantage’ (including but not limited to disadvantaged communities, poverty, neighbourhood/area status); or any definition of ‘individually measured disadvantage’ (including but not limited to low income, entitlement to medical or other state benefits, unemployment, educational status and social class);
(iv) included both men and women if findings were reported separately for women or were available for women;
(v) included both pregnant and non-pregnant women if smoking cessation was reported separately or available for non-pregnant women; and
(vi) included women living in any circumstances where results of women in disadvantaged communities were reported separately or were available.

Studies were excluded if (i) they were exclusive to men; (ii) included only pregnant women; (iii) were not available in English; or (iv) used quasi-experimental methodologies. No limitations were placed on dates. Only peer-reviewed, published studies were included. Database searches occurred in April 2019.

This study is registered with PROSPERO (registration ID: CRD42019130160) and is available online [36].

### Procedure

All retrieved citations were imported into Covidence® (Cochrane, Melbourne, Australia) and duplicates were removed. Two reviewers (NOC and EB) independently reviewed all titles and abstracts. Differences concerning inclusion were resolved through consensus. Upon completion of title reviews, two reviewers (NOC and FD) screened the full text of all studies. Differences concerning the inclusion of 31 full texts (26.7% of total) were arbitrated by a third reviewer (CBH).

NOC independently extracted data from all studies and FD extracted from a sample of studies to ensure there was consistency of approach using Covidence. There was full consistency in the data extraction of both authors. We were guided by the TIDieR checklist in the reporting of study interventions [37].

### Data analysis

We collected the following information on low-SES women only: SES definition; ethnicity; setting; intervention type and content; intervention duration provider and associated provider training; intervention fidelity; comparison arms; length of study follow-up and outcome measures; and intervention completion rates.

Where cluster randomisation was used, we assessed whether authors accounted for the intra-class correlation (ICC). ICC estimates the relative variability within and between clusters. Where the ICC was not reported, we assumed an ICC of 0.013 as this ICC was reported in two previous smoking intervention cluster RCTs [38, 39]. The design effect was calculated as 1 + ((M – 1)*ICC), where M is the average cluster size and the number of participants and number experiencing the event should be divided by the design effect [40].

Abstinence rates were summarised as risk ratios (RRs) with 95% confidence intervals (CIs) which were calculated using the following formula: ([number of quitters: intervention arm]/[number randomised: intervention arm])/([number of quitters: control arm]/[number randomised: control arm]). While our protocol states that we would calculate odds ratios, risk ratios were used instead as they are typically a more clinically understood effect measure [40]. Descriptive data is displayed alongside RRs with 95% CIs in forest plots.

We assessed statistical heterogeneity using the *I*^*2*^ statistic which assesses the proportion of variation between studies due to heterogeneity rather than chance [41]. *I*^*2*^ values range from 0 to 100%, with 0% indicating no heterogeneity and larger values indicating greater heterogeneity. Values of 25%, 50% and 75% correspond to low, moderate and high levels of statistical heterogeneity respectively [41].

We assessed studies for missing data. Missing data were not reported in two studies. A sensitivity analysis, excluding both studies did not alter the results. Both studies were therefore retained in the meta-analysis. Where statistical heterogeneity and measures of inconsistency were acceptable, we conducted a meta-analysis to compare intervention and control cessation rates at the earliest available end point in each study (end-of-treatment). We conducted a second meta-analysis which compared intervention and control group smoking cessation at the latest available follow-up point and only in instances where a time period had elapsed since end-of-treatment. We conducted intention-to-treat (ITT) analyses in each meta-analysis; hence, all randomised participants were included in the denominator and individuals lost to follow-up were counted as smokers. Studies were weighted by the inverse variance of their effect size estimates.

We conducted four ’subgroup comparisons’ to further explore heterogeneity using forest plots. These included comparisons of (1) interventions targeting only women versus interventions including men and women; (2) interventions using biochemical verification as part of their smoking cessation outcome measure versus those that did not; (3) interventions taking place in clinical settings versus any other setting; (4) interventions comparing face-to-face support (e.g. motivational interviewing) with any other intervention type (e.g. telephone support); and interventions which used pharmacotherapy versus those that did not. Publication bias was assessed using a funnel plot.

Review Manager software (RevMan, Version 5.3. Copenhagen: The Nordic Cochrane Centre, The Cochrane Collaboration, 2014) was used to conduct the meta-analysis. Random-effects (RE) methods for dichotomous data were selected using the Mantel-Haenszel (M-H) method [40]. The expected clinical diversity and range of outcome timepoints reported in the trials warranted selection of an RE model as we could not assume the meta-analysis would estimate a common underlying effect.

### Risk of bias

We assessed methodological quality of eligible studies using the Cochrane Risk of Bias 2 tool [42] to assess the following biases: those arising from the randomization process; due to deviations from the intended intervention; missing data; measurement of the outcome, and bias in selection of the reported result [43].

### GRADE approach

The GRADE (Grades of Recommendation Assessment, Development and Evaluation) approach was used to assess the overall certainty of the evidence for each outcome [44]. The approach classifies the certainty of the evidence into high, moderate, low and very low levels. An RCT starts as high-certainty evidence and the certainty of evidence is downgraded according to five domains: risk of bias; inconsistency; indirectness; imprecision; and publication bias [45].

GRADE Pro software was used for this assessment (GRADEpro GDT: GRADEpro Guideline Development Tool [Software]. McMaster University, 2015).

Risk of bias and GRADE assessments were performed by NOC under the supervision of CBH.

### Ethics

Ethical approval was not required for this systematic review.

## Results

**Fig. 1:**
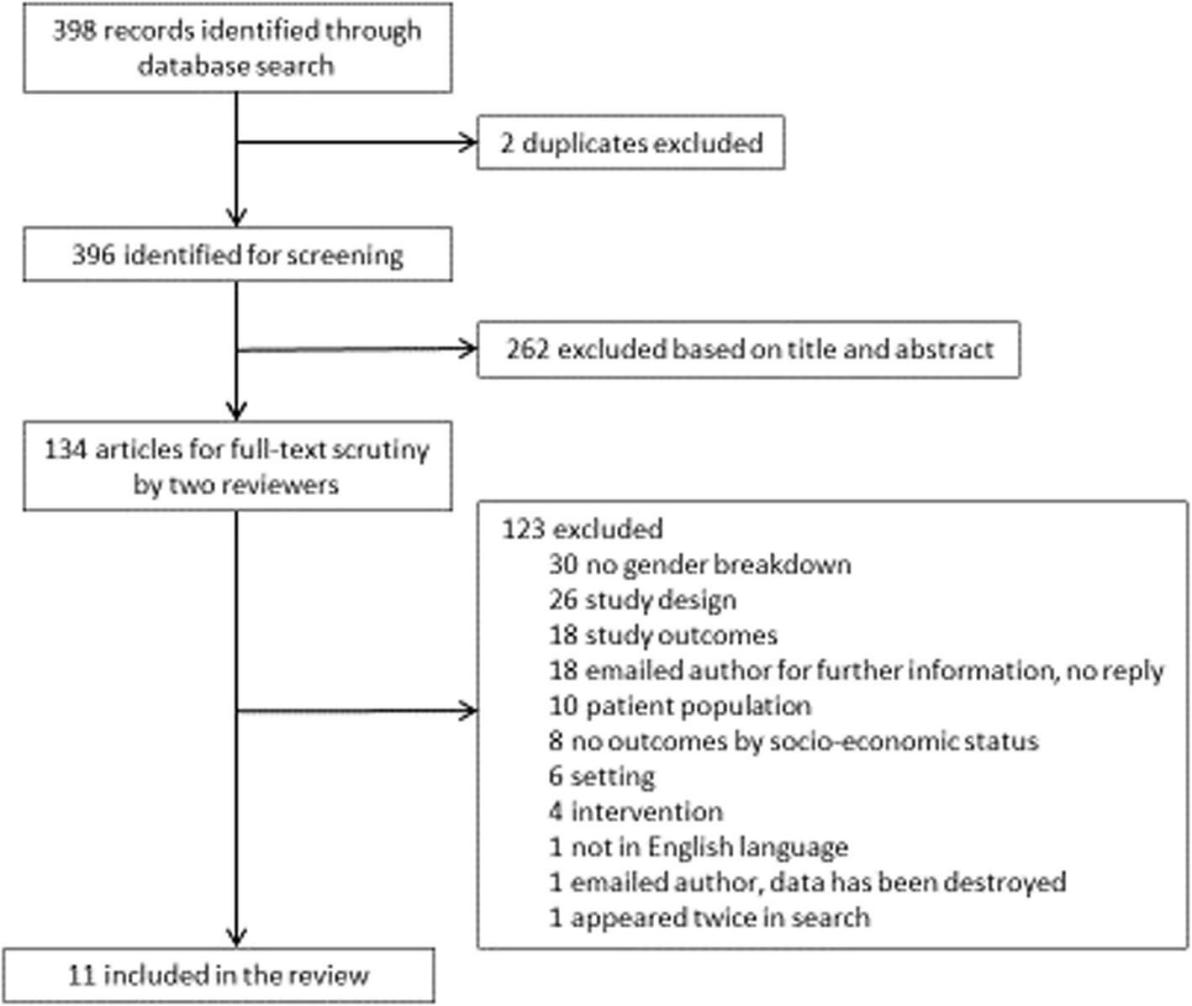
PRISMA flow diagram

Figure 1 displays the systematic review PRISMA flow chart. Of 398 studies retrieved and screened for eligibility, in total, 27 studies were chosen for inclusion in the systematic review. Following the commencement of data extraction, five additional studies were excluded. Of these five studies, two did not provide relevant information for determining SES status of included participants; one was a protocol; and one included non-smokers in the analysis. The fifth reported limited trial outcomes and the author advised the main trial paper would not be published until 2020. A further 13 studies did not contain sufficient outcome information necessary for data extraction. Authors of these 13 studies were contacted and three responded. Two authors supplied the required information [46, 47] and one was unable to provide further information as the data were previously destroyed. In total, 11 studies were included. Table 1 describes the 11 included studies in detail. The PRISMA checklist is included (see Supplementary Material 2).

**Table 1:** Study characteristics

	Origin	Study design	Setting	For women only?	No. of women randomised (% total randomised)	Tailored for low SES?	Mean age, years	Baseline cigarettes per day, mean	Intervention condition	Control condition	Pharmaco-therapy offered to intervention group?	Outcome	Length of intervention ((post randomisation)	Follow-up (post-randomisation)	Bio-chemical verification	SES measure
Andrews et al. 2016 [48]	USA	Two-group pragmatic cluster RCT	Subsidised neighbourhoods	Yes	409 (100%)	Yes	42	12.7	Face-to-face individual, group support, NRT, neighbourhood anti-smoking activities	Written materials	NRT	7-day point prevalence	6 months	6 months, 12 months	Yes	Residence in public housing neighbourhood
Bernstein et al. 2015 [47]	USA	Two-group RCT	Urban emergency department	No	407 (52.2%)	Yes	No female only info	No female only information	Brief face-to-face motivational interviews, referral to phoneline, NRT	Written materials and phone line number	NRT	7-day point prevalence	3 months	3 months	Yes	Receipt of Medicaid or held no insurance
Collins et al. 2019 [49]	USA	Two-group RCT	Paediatric primary care community clinics and community	Yes	300 (100%)	Yes	29.4	12.3	Face-to-face and phone motivational interviews, written material	Brief advice, written material	No	7-day point prevalence	4 months	4 months	Yes	Low-income, racial minority communities
Curry et al. 2003 [50]	USA	Two-group RCT	Paediatric clinics serving ethnically diverse populations of low-income families	Yes	303 (100%)	Yes	34	12.1	Face-to-face motivational interviewing, telephone support, written material	Usual care	No	7-day point prevalence	3 months	3 months, 12 months	No	Ethnically diverse population of low-income families
Etter et al. 2016 [51]	Switzerland	Two-group RCT	Low-income smokers in the general population	No	415 (51.4%)	Yes	No female only info	No female only information	Signed ‘contract to quit’, social supporter designated (friend/family), written materials, website access and escalating financial incentives (gift cards)	Written materials and website access	No	Continued abstinence between 6 and 18 months	6 months	6 months	Yes	Income ≤ 50,000 Swiss francs (CHF) ($55,000, single) or CHF ≤ 100,000 ($110,000, married).
Gilbert et al. 2017 [46]^a^	England, UK	Two-group RCT	NHS Stop Smoking Services and general practices in England	No	Women in high deprivation1075 (24.5) All women in RCT 2152 (49.1)	No	All women in sample (not just low SES): 48.0	Not available	Tailored, personalised letter from GP to attend ‘Come and Try It’ taster, repeated letter 3 months later	Generic letter from GP advertising local Stop Smoking Service and available therapies	No	7-day point prevalence	Letter sent at start of study and reminder sent at 3 months	6 months	Yes	Index of Multiple Deprivation score (IMD), official measure of multiple deprivations at the small-area level.
Glasgow et al. 2000 [52]	USA	Two-group RCT	4 Planned Parenthood clinics	Yes	1154 (100%)	Yes	24	12	Brief behavioural support, clinician advice, written materials, telephone support.	Written materials and advice	No	30-day point prevalence	6 weeks	6 weeks, 6 months	Yes	Clinics serves low-income women
Haas et al. 2015 [53]	USA	Two-group RCT	Primary care practices in the Greater Boston area (6 community health centres, 2 community-based practices, 4 hospital-based practices, and 1 medical home)	No	482 (68.2%)	Yes	No female only information	No female only information	Motivational interviewing telephone support and NRT	Usual care	NRT	7-day point prevalence	1 ½ months	9 months	No	Residence in a low (<$45 000) or moderate ($45 000-$67 050) median household income census tract
Manfredi et al. 2004 [54]	USA	Two-group cluster RCT	Prenatal, family planning and paediatric services in public health clinics in Chicago and two suburbs	Yes	1068 (100%)	Yes	29	11	Telephone support, reminder letter, posters, video, advice, written materials and signature on patient-provider agreement form.	Advice, written materials, posters, video	No	7-day point prevalent abstinence	5–8 weeks	2 months, 6 months, 12 months and 18 months	No	Attendance at public health clinic
Solomon et al. 2000 [55]	USA	Two-group RCT	Community	Yes	214 (100%)	Yes	33	23.7	NRT, telephone support	NRT	NRT	7-day point prevalent abstinence	3 months	3 months and 6 months	Yes	Medicaid-eligible
Solomon et al. 2005 [56]	USA	Two-group RCT	Community	Yes	330 (100%)	Yes	34.2	23.6	NRT, telephone support	NRT	NRT	7-day point prevalent abstinence	4 months	3 months and 6 months	No	Medicaid-eligible

^a^Data from Gilbert’s study is derived from information the author sent upon request. This was broken down by gender and the UK’s Multiple Deprivation Score. We use female-only data from IMD quintiles 4 and 5 only, areas classified as ‘high deprivation’

All studies were two-group RCTs, of which two were cluster RCTs [48, 54]. The total number of women in low-SES groups randomised to all 11 interventions was 6153. Of these, 3190 women were randomised to intervention groups and 2963 to control groups.

Seven of the 11 studies targeted women only and four included men [46, 47, 51, 53]. One study was not specifically tailored to low socio-economic groups [46]; however, the authors provided RCT results of women living in high deprivation areas and these results are included in this review and meta-analysis.

### SES

Measures of SES varied across studies from individual-level to area-level definitions (Table 1). Individual-level definitions were used in six studies. These were: receipt of or eligibility for Medicaid (a federal and state programme which assists with medical costs for people with limited income and resources) [47, 55, 56], residence in public housing [48] and specific income thresholds [51, 53]. Area-level definitions of low-SES included people living in low-income and racial minority communities [49, 50], small area deprivation [46] and attendance at clinics serving low-income women or a public health clinic [52, 54].

### Ethnicity

Six studies reported ethnicity rates [47–50, 52, 54]. Of these, the number of non-White women including Black, Hispanic and Asian females as a proportion of the total women randomised in those studies was 2511 (58.5%). African American women were the most common ethnicity in five of the six studies [47–50, 54].

### Setting

Nine studies were based in the USA [47–50, 52–56], one in Switzerland [51] and one in England [46].

Seven recruited patients through screening within emergency departments [47], paediatric primary care and community clinics [49, 50], primary care [46, 53], family planning [52] and public health clinics [54] (Table 1). Other studies used a combination of information stands, flyers, advertising within the community, advertising on the internet and in the press and on public bulletin boards [48, 55, 56]. In one, community advertising was displayed in the press, online, and within workplaces, pharmacies and medical and dental clinics [51].

### Intervention type and content

Five interventions tested a form of face-to-face individual support such as motivational interviewing or brief behavioural support [47–50, 52] (Table 1). Only one study provided a group intervention [48]. Telephone support was provided in combination with a face-to-face intervention in four studies [47, 49, 50, 52]. In a further four studies, telephone support was provided without any face-to-face intervention [53–56]. NRT was offered in conjunction with other forms of support in five studies [47, 48, 53, 55, 56] and written materials were offered in four [46, 49, 51, 54]. Etter et al. [51] tested the signing of a ‘contract to quit’ alongside the designation of a friend or family member as a ‘social supporter’. Gilbert et al. [46] tested the efficacy of a personalised letter sent from a GP inviting smokers to a smoking cessation taster session with a reminder letter 3 months later.

The interventions targeted a range of behavioural change processes (see Table 2). Andrews et al. [48] targeted both group (educational and behavioural strategies) and individual-level (social support and self-efficacy) processes. Developing self-efficacy, self-confidence, providing encouragement and positive reinforcement were behavioural targets in interventions by Solomon et al. [55], Solomon et al. [56], Bernstein et al. [47], Curry et al. [50] and Haas et al. [53]. Provision of advice, guidance and addressing lack of knowledge were targeted by Bernstein et al. [47], Gilbert et al. [46], Solomon et al. [55] and Solomon et al. [56]. Improving motivation and readiness to quit and reducing smoking cessation ambivalence were targeted by Glasgow et al. [52], Haas et al. [53] and Manfredi et al. [54]. Collins et al. [49] targeted the management of smoking urges and support for parents to help protect children from secondary smoke exposure and Etter et al. [51] targeted sustained abstinence, rather than initial quit attempts.

**Table 2:** Intervention characteristics

	Theoretical framework and/or rationale	Behavioural targets of intervention	Intervention deliverer and training provided	Intervention fidelity measures
Andrews et al. 2016 [48]	Community-based participatory research approach and social ecological model	**Group**: educational and behavioural strategies **Individual**: social support with quitting and enhanced self-efficacy with cessation attempts	**Group strategies**: certified counsellors and neighbourhood tenant association **Individual**: community health workers	Fidelity observation checklist for community health worker, peer group, neighbourhood, written materials, patches implemented
Bernstein et al. 2015 [47]	Low-income smokers have limited access to GP services who undertreat smoking. ED visit opportunity for screening, intervention and referral	Feedback, enhancement of self-efficacy, brief advice and treatment options given in non-judgemental, empathic fashion to improve self-reflection	**Interviews**: research associates trained in motivational techniques **NRT:** ED nurse	All interviews audio-recorded and reviewed weekly with research associates by psychologist
Collins et al. 2019 [49]	Behavioural counselling suggests social continencies restrict residential smoking	Identifying and managing urges to smoke and building support to protect children from TSE	Clinical social workers and graduate students Training by doctoral-level experts in smoking cessation	Audio-taped assessment interviews and counselling sessions. Supervision by smoking cessation experts included review of cases and fidelity to maintain >90% fidelity with intervention protocol
Curry et al. 2003 [50]	The paediatric clinic as a ‘teachable setting’ in which to provide advice and assistance to parents who smoke	Helping smokers articulate concerns about smoking and reasons for quitting. **Motivational interviewing**: aim to trigger decision and commitment to change through feedback, enhancing personal responsibility, advice and supporting self-confidence by using the success of others as encouragement, within a non-confrontational and empathic context.	Motivational message from child’s clinician. Motivational interview and phone call from clinic nurse or study interventionist	Regular review of the visit and call summary sheets by project director, biweekly supervision by telephone, and quarterly in-person lunch meetings
Etter et al. 2016 [51]	Low-income smokers are hard to reach. Financial incentives should be high enough to compensate for tobacco withdrawal symptoms and loss of a valued activity.	Rewarding sustained abstinence, rather than initial quit attempts	Research assistants with no training in smoking cessation support	Not stated
Gilbert et al. 2017 [46]*	1. Direct marketing and proactive recruitment (e.g. cold-calling) has potential as recruitment strategy for smokers. 2. Enhancing personal relevance can help tailor messages to the recipient (computer-tailoring) 3. 3Ts model (tension, trigger, treatment)–inform smoker of personal risk, promote confidence and provide helping relationship	Addresses lack of knowledge or inadequate information on available stop smoking services. Use of ‘why quit’ messages, hard-hitting messages about the consequences of tobacco use and ‘how to quit’ messages, supportive and positive and emphasising quitting resources	Letters generated by a research assistant in each primary care practice	Research assistants trained in RCT methods emphasising importance of standardising taster sessions, and delivering all protocol-specified content, while allowing for differences in the organisation of the individual Stop Smoking Services. Taster sessions were audio-recorded. Advisors completed a personal details form, gathering personal data and highest educational qualification, type of smoking cessation training, time since smoking cessation training, number of patients seen in the previous 6 months and job title to account for differences in ‘therapist effects’.
Glasgow et al. 2000 [52]	Lower SES women have multiple barriers to participation in smoking interventions. Planned parenthood clinics many low-income women and are important setting	Motivational interviewing and barrier-based counselling. Personalized strategies used based on readiness to quit and barriers to quitting	Planned Parenthood clinical staff who received a 1-h training session	Delivery of intervention components was measured and reported—no other details stated
Haas et al. 2015 [53]	GPs do not have time or training to provide tobacco treatment. 1. Dissemination of electronic health records with smoking status data is tool to reach smokers. 2. Interactive voice response allows a computer to detect voice responses during a call and is efficient means to reach large population can be used to engage smokers by providing ink to tobacco specialist	Motivational interviewing techniques to help resolve ambivalence about behaviour change regardless of readiness-to-quit standard. Content tailored to the individual based on intent and confidence to quit. Participants could select optional modules based on needs (e.g. stress, weight gain, menthol use)	Tobacco treatment specialist	Not assessed
Manfredi et al. 2004 [54]	Transtheoretical model of stages of change, social marketing, social learning and motivation theories, self-help for quitting strategies	Improving motivation and readiness to quit smoking in addition to helping smokers ready to quit	Clinical personnel	Not stated
Solomon et al. 2000 [55]	Focuses on low-income women of childbearing age where smoking prevalence is high and cessation resources are limited	Encouragement, guidance and reinforcement for quitting smoking and helped women cope with high risk for smoking situations. They negotiated a schedule of contact	Support person	Brief quality control checks conducted by phone by research assistant with sample of group
Solomon et al. 2005 [56]	Repeat of their 2000 study but provides longer and more intensive telephone contact to see if abstinence is improve at 6 months	A semi-structured protocol designed to provide encouragement, guidance and reinforcement for quitting smoking, and to assist the woman in problem-solving high-risk-for-smoking situations.	Support person who received periodic refresher training sessions and telephone contact to review and discuss protocol	Support logs submitted to author for review each month. Quality control on 50% of participants to verify contact and ensure calls were well-received.

### Intervention duration, provider and provider training

Table 1 outlines the duration of each intervention. Interventions varied in length between 1.5 [53] and 6 months [48, 51]. Three studies tested 3-month interventions [47, 50, 55] and two tested 4-month interventions [49, 56]. The intervention by Glasgow et al. [52] was 6 weeks in duration and Manfredi et al.’s [54] between 5 and 8 weeks. Gilbert et al. [46] tested the once-off sending of a letter with a 3-month follow-up letter sent if the receiver did not respond to the first letter.

Mode of intervention delivery varied (see Table 2). Three studies tested interventions delivered by trial Research Assistants [46, 47, 51]. In addition, three service clinicians delivered the interventions [50, 52, 54]. In five studies, the interventions used specific clinical specialists who were not based within a service. Andrews et al. [48] employed certified smoking cessation counsellors for behavioural group sessions and community health workers for one-to-one contacts, Collins et al. [49] used clinical social workers, Haas et al. [53] used tobacco treatment specialists and Solomon et al. [55] and Solomon et al. [56] employed support workers.

Six studies described the training intervention providers received; however, information varied on the level and type of training provided (Table 2). Bernstein et al. [47] trained Research Associates in motivational techniques. Collins et al. [49] provided training by doctoral-level experts in smoking cessation. Gilbert et al. [46] trained Research Assistants in RCT methodology. In Glasgow et al. [52], Planned Parenthood staff received a 1-h training session whereas in Solomon et al. [56], support staff received periodic refresher training. The intervention by Etter et al. tested the delivery of financial incentives [51] and the Research Assistants delivering the incentives were not trained in smoking cessation techniques.

### Intervention fidelity

Measurements of fidelity varied between studies (Table 2). Manfredi et al. [54] and Etter et al. [51] did not state whether any fidelity checks occurred. Haas et al. [53] stated fidelity was not assessed and Glasgow et al. [52] write that intervention delivery was measured, but provide no further detail. Andrews et al. [48] used a fidelity checklist. Supervision by experts was provided by Collins et al. [49]. Curry et al. [50], Bernstein et al. [47] and Gilbert et al. [46] audio-recorded interviews. Solomon et al. [56] and Curry et al. [50] reviewed call summary sheets and call logs. Solomon et al. [56] and Solomon et al. [55] conducted quality control calls with participants to verify contact and to ensure calls were well received.

### Comparison arms

Two studies compared the intervention to usual care [50, 53] and four used single control comparators (Table 1). These included written materials [46, 48] and NRT [55, 56]. A combination of control conditions were allocated in the remaining studies.

### Outcome measures and periods of follow-up

Studies reported cessation outcomes at multiple endpoints (Table 1). All studies used 7-day point prevalence abstinence as the primary outcome measure, with the exception of Etter et al. [51] and Glasgow et al. [52] who used ‘continued abstinence between 6 and 18 months’ and ’30-day point prevalence’ respectively. Biological verification through carbon monoxide or urine cotinine samples was collected in seven studies, but was not used by Curry et al. [50], Haas et al. [53], Manfredi et al. [54] or Solomon et al. [56].

Participant follow-up dates differed (Table 1). The longest follow-up period post-randomisation was 18 months [54], while the shortest was 3 months, which coincided with end of treatment [47].

### Process measures

Three studies did not report process measures [47, 51, 53] (Table 3). In studies with available data, intervention completion rates were reasonably high. The highest proportion of women completing the intervention was 95%, reported by Solomon et al. [55]. The lowest number reported was in Collins et al. [49] with 105 completing the intervention of the 145 (72.4%) randomised.

**Table 3:** Process outcomes: outcome completion rates and intervention attendance

	No. of women randomised (% total randomised)	No. of women randomised to intervention (% women randomised)	No. of women randomised to control (% women randomised)	No. of women who completed intervention (% randomised to intervention)	No. of women who completed control (% randomised to control)	No. of intervention women who completed 1^**st**^ follow-up (% randomised to intervention)	No. of control women who completed 1^**st**^ follow-up (% randomised to control)	No. of intervention women who completed subsequent follow-up (% randomised to intervention)	No. of control women who completed subsequent follow-up (% randomised to control)	Number of intervention sessions offered	Number of intervention sessions attended
Andrews et al. 2016 [48]	409 (100)	200 (48.9)	209 (51.1)	189 (94.5)	192 (91.9)	12 months: 185 (92.5)	12 months: 188 (89.95)	NA	NA	- 16 planned community health worker (CHW) visits - 6 behavioural group sessions	- Mean visits/participants: 11.2 of 16 - Mean group sessions attended: 4 of 6 - 133 used nicotine patch for average of 2.8 weeks.
Bernstein et al. 2015 [47]	407 (52.2)	218 (53.6)	189 (46.4)	No female-only data	No female-only data	No female-only data	No female-only data	No female-only data	No female-only data	No female-only data	No female-only data
Collins et al. 2019 [49]	300 (100)	145 (48.3)	155 (51.7)	105 (72.4)	124 (80)	NA	NA	NA	NA	- 10 (2 home visits, seven phone sessions, and one quit day phone session if mother set a quit day)	- 104 (71.7%) completed 8–10 sessions - 16 (11%) completed 5–7 sessions - 9 (6.3%) completed 2–4 sessions - 16 (11%) completed 1 session
Curry et al. 2003 [50]	303 (100)	156 (51.5)	147 (48.5)	120 (76.9)	121 (82.3)	12 months: 121 (77.5)	12 months: 123 (83.7)	NA	NA	- 1 motivational message with physician - 1 motivational interview with nurse - Up to 3 telephone calls	- 68% discussed smoking with child’s physician - 74% received motivational interview with nurse - 78% received at least one telephone call
Etter et al. 2016 [51]	415 (51.4)	189 (45.5)	226 (54.5%)	No female-only data	No female-only data	No female-only data	No female-only data	No female-only data	No female-only data	No female-only data	No female-only data
Gilbert et al. 2017 [46]^a^	1075 (24.5)	633 (58.9)	442 (41.1)	466 (73.6)	332 (75.1)	NA	NA	NA	NA	- Invitation to Taster session run by Stop Smoking Services - Attended SSS sessions (6 in total)	- 137/633 (21.6%) attended 1 Taster session - 69/633 (10.9%) attend 6 sessions
Glasgow et al. 2000 [52]	1154 (100)	578 (50.1)	576 (49.9)	536 (92.7)	536 (93.1)	6 months: 502 (86.9)	6 months: 531 (92.2)	NA	NA	- Video - Counselling - Provider advice - Telephone calls	- 85% saw video - 92% received counselling - 95% received provider advice - 42% received one call or more; 11% received two or more calls
Haas et al. 2015 [53]	482 (68.2)	271 (67.9)	211 (68.5)	No female-only data	No female-only data	No female-only data	No female-only data	No female-only data	No female-only data	No female-only data	No female-only data
Manfredi et al. 2004 [54]	1068 (100)	527 (49.3)	541 (50.7)	456 (86.5)	506 (93.5)	6 months: 392 (74.4)	6 months: 470 (86.9)	12 months: 293 (55.6) 18 months: 226 (42.9)	12 months: 361 (66.7) 18 months: 285 (52.7)	Information unavailable	Information unavailable
Solomon et al. 2000 [55]	214 (100)	106 (49.5)	108 (50.5)	101 (95)	92 (85)	6 months: 77 (73)	6 months: 79 (73)	NA	NA	- Support calls - NRT	- 97/101 (96%) reported use of NRT - 96/101 (95%) received one or more support calls (mean of 7 calls over 3 months)
Solomon et al. 2005 [56]	330 (100)	171 (51.8)	159 (48.2)	161 (94.2)	147 (92.5)	6 months: 149 (87)	6 months: 138 (87)	NA	NA	- Support calls - NRT	- 157/161 (98%) reported used of NRT - 158/161 (98%) received one or more support calls

^a^We report data on women living in high deprivation areas only rather than the total female sample

In studies that provided follow-up data beyond the end of the intervention, follow-up rates dropped in most (see Table 3). For participants randomised to intervention arms, the highest follow-up rate was Andrews et al.’s [48] Sister-to-Sister intervention with 185/200 (92.5%) women randomised responding at 12-month follow-up. The lowest rate of 226/456 (42.9%) was reported by Manfredi et al. [54]. Data on intervention attendance were reported differently in each trial. The highest attendance rates were reported in both trials by Solomon et al. [55, 56].

### Meta-analysis

Outcomes from 10 of the 11 studies were combined in each meta-analysis. One study by Haas et al. [53] was excluded prior to meta-analysis consideration as it did not publish the number or proportion of abstinent smokers.

The remaining 10 studies were assessed for statistical heterogeneity and inconsistency. The *I*^2^ value was 34%, and therefore deemed within acceptable limits to proceed to a meta-analysis [40]. Each study specified a greater than 50% follow-up for outcome measures; however, missing data for females were not reported in two studies [47, 51] (see Table 3).

Two of the trials were cluster RCTs but neither reported whether the intra-class correlation (ICC) had been accounted for in the original analysis [48, 54]. Please see Supplementary Material 3 for a description of how we accounted for the design effect in the Andrews et al. [48] and Manfredi et al. [54] studies.

### Primary outcome

For trial related end-of-treatment smoking cessation outcomes, or the first available follow-up, we found a low level of statistical heterogeneity (chi^2^ = 13.66; degrees of freedom = 9; *p* = 0.13), and low ‘inconsistency’ in the effect size between trials (*I*^*2*^ = 34%).

**Fig. 2:**
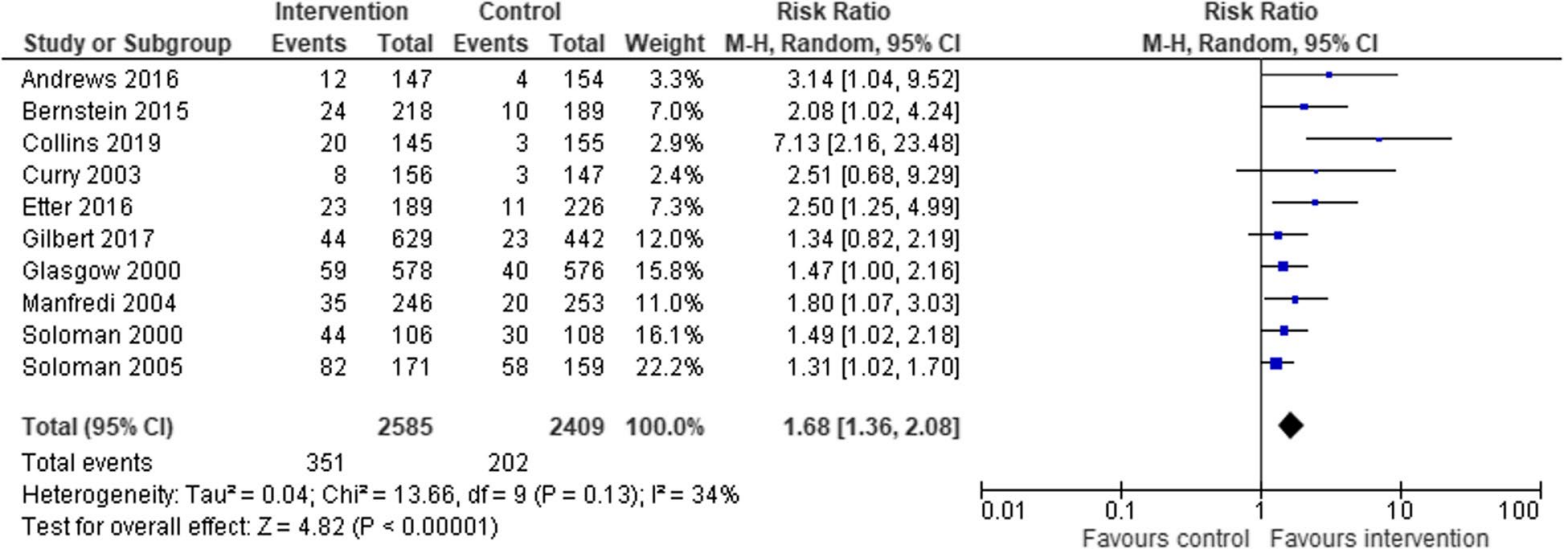
Smoking cessation in intervention participants versus control participants at end of intervention or at earliest available end point

Analyses with data from 10 studies showed that women in low-SES positions, who received a smoking cessation intervention (*n* = 2585) were statistically significantly more likely to have stopped smoking than women in low-SES positions in control groups (*n* = 2409) (RR 1.68, 95% CI 1.36–2.08; *I*^*2*^ = 34%) (Fig. 2).

### Outcome at further follow-up

Six studies completed further follow-ups after the end of intervention. We conducted a meta-analysis using the latest available follow-up data in trials where a period of time had passed between end-of-treatment and the follow-up (median = 9 months, interquartile range = 6–13.5 months). Using the same design effect size, we recalculated outcomes and sample sizes in the two cluster RCTs [48, 54]. The intervention event size was reduced from 18 to 13 in Andrews et al., and control events were reduced from 9 to 7. In Manfredi et al, the intervention event size reduced from 59 to 28 and the control event size reduced from 69 to 32.

**Fig. 3:**
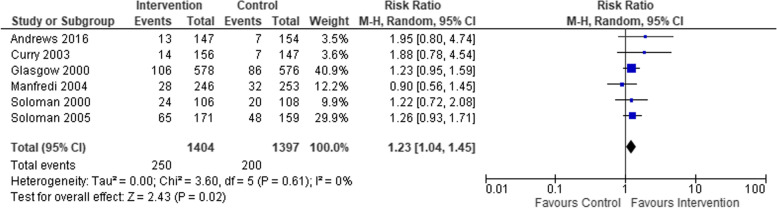
Smoking cessation outcomes in studies with time points available beyond end-of-intervention

The difference in cessation rates between intervention (*n* = 1404) and control (*n* = 1397) groups remained statistically significant, however, the RR was reduced (RR 1.23, 95% CI 1.04–1.45) (see Fig. 3). Statistical heterogeneity was not present (Chi^2^= 3.60; degrees of freedom = 5; *p* = 0.61) and *I*^*2*^ was low (*I*^*2*^*=* 0%).

### Risk of bias

Most studies were considered to have some degree of bias (‘some concerns’) as authors did not specify whether statistical analyses were pre-defined (Table 4). In five studies, a high risk of bias was indicated due to the use of self-report rather than biological verification in the smoking cessation outcomes.

**Table 4:** Risk of bias assessments

Studies	Bias arising from the randomisation process	Bias due to deviations from intended interventions	Bias due to missing outcome data	Bias in measurement of the outcome	Bias in selection of the reported result	Overall bias
Andrews et al. 2016 [48]	Low risk	Low risk	Low risk	Some concerns: no information on blinding of outcome assessors	Some concerns: no information on whether statistical analysis was pre-planned	Some concerns
Bernstein et al. 2015 [47]	Some concerns: Some differences in baseline characteristics between groups	Low risk	Low risk	Low risk	Low risk	Some concerns
Collins et al. 2019 [49]	Low risk	Low risk	Retention rate relatively low		High risk: authors changed statistical analysis plan due to low retention rate^a^	High risk
Curry et al. 2003 [50]	Low risk	Low risk	Low risk	High risk: no biochemical validation in primary outcome	Some concerns: no information on whether statistical analysis was pre-planned	High risk
Etter et al. 2016 [51]	Some concerns: Some differences in characteristics between groups, but accounted for in analysis	Low risk	Low risk	Low risk	Some concerns: No information on whether statistical analysis was pre-planned	Some concerns
Gilbert et al. 2017 [46]	Low risk	Low risk	Low risk	Low risk	Low risk	Low risk
Glasgow et al. 2000 [52]	Low risk	Low risk	Low risk	Some concerns: no information on blinding of outcome assessors	Some concerns: no information on whether statistical analysis was pre-planned	Some concerns
Haas et al. 2015 [53]	Low risk	Low risk	Some concerns: Retention rate relatively low and higher assessment rate in control group	High risk: no biochemical validation in primary outcome	Some concerns: no information on whether statistical analysis was pre-planned	High risk
Manfredi et al. 2004 [54]	Some concerns: differences in racial characteristics between groups	Low risk	Some concerns: Retention rates low, but equally low in both groups	High risk: no biochemical validation in primary outcome	Some concerns: no information on whether statistical analysis was pre-planned	High risk
Solomon et al. 2000 [55]	Low risk	Low risk	Low risk	Some concerns: no information on blinding of outcome assessors	Some concerns: no information on whether statistical analysis was pre-planned	Some concerns
Solomon et al. 2005 [56]	Low risk	Low risk	Low risk	High risk: no biochemical validation in primary outcome	Some concerns: no information on whether statistical analysis was pre-planned	High risk

^a^The authors explain the change in analyses as “We planned to test the interaction between treatment and other smokers in home at *p*<0.05 when initial models demonstrated main effects of both variables. Although each of the predictors and outcome variables contained small numbers of missing values, an analysis of complete data only would have reduced our sample by about one third. To retain our sample and avoid bias arising from missing data, we used multiple imputation methods, which also estimate SEs that incorporate the uncertainty due to imputation

### GRADE

**Fig. 4:**
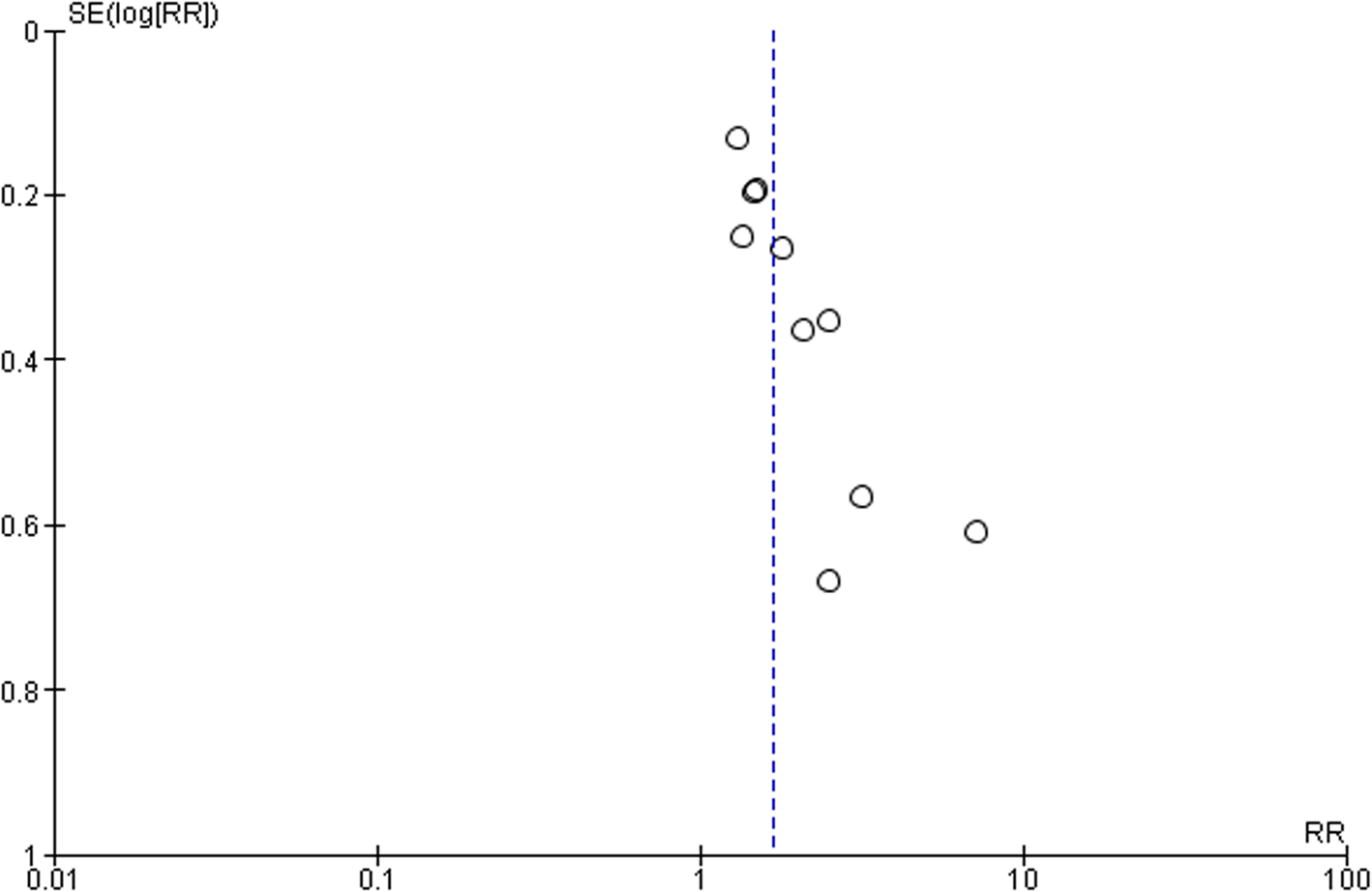
Funnel plot showing risk of publication bias. The y-axis represents study precision (standard error) and the *x*-axis represents individual risk ratios. This asymmetric funnel plot suggests the presence of publication bias

The certainty of evidence according to GRADE was low (Table 5) [40]. In addition to the risk of bias noted above, there was a potentially serious risk of imprecision due to the small sample sizes of the included RCTs, despite the presence of a large intervention effect, and a strong suspicion of publication bias suggested by presence of an asymmetric funnel plot (Fig. 4).

**Table 5:** GRADE summary of findings table

**Smoking intervention compared to control condition for smoking cessation in socio-economically disadvantaged women**					
**Patient or population**: Smoking cessation in socio-economically disadvantaged women or women living in disadvantaged areas **Setting**: Varied **Intervention**: Smoking intervention **Comparison**: Control condition					
**Outcomes**	**№ of participants (studies) Follow-up**	**Certainty of the evidence (GRADE)**	**Relative effect (95% CI)**	**Anticipated absolute effects**	
				**Risk with control condition**	**Risk difference with smoking intervention**
Smoking cessation at intervention end	5671 (10 RCTs)	⨁⨁◯◯ LOW ^a,b,c^	**RR 1.67** (1.45 to 1.94)	82 per 1000	**55 more per 1000** (37 more to 77 more)
*The risk in the intervention group (and its 95% confidence interval) is based on the assumed risk in the comparison group and the **relative effect** of the intervention (and its 95% CI). **CI:** confidence interval; **RR:** risk ratio					
**GRADE working group grades of evidence** **High certainty:** We are very confident that the true effect lies close to that of the estimate of the effect **Moderate certainty:** We are moderately confident in the effect estimate: the true effect is likely to be close to the estimate of the effect, but there is a possibility that it is substantially different **Low certainty:** Our confidence in the effect estimate is limited: the true effect may be substantially different from the estimate of the effect **Very low certainty:** We have very little confidence in the effect estimate: the true effect is likely to be substantially different from the estimate of effect					

^a^The risk of bias table suggests there is some concern in most studies, with high risk of bias in 5 of the 11 studies

^b^Imprecision: the number of events for each outcome was less than 300 in all studies except Gilbert, Glasgow and Manfredi although the effect size is large

^c^The funnel plot shows asymmetry suggesting the presence of publication bias

### Subgroup comparisons

**Fig. 5:**
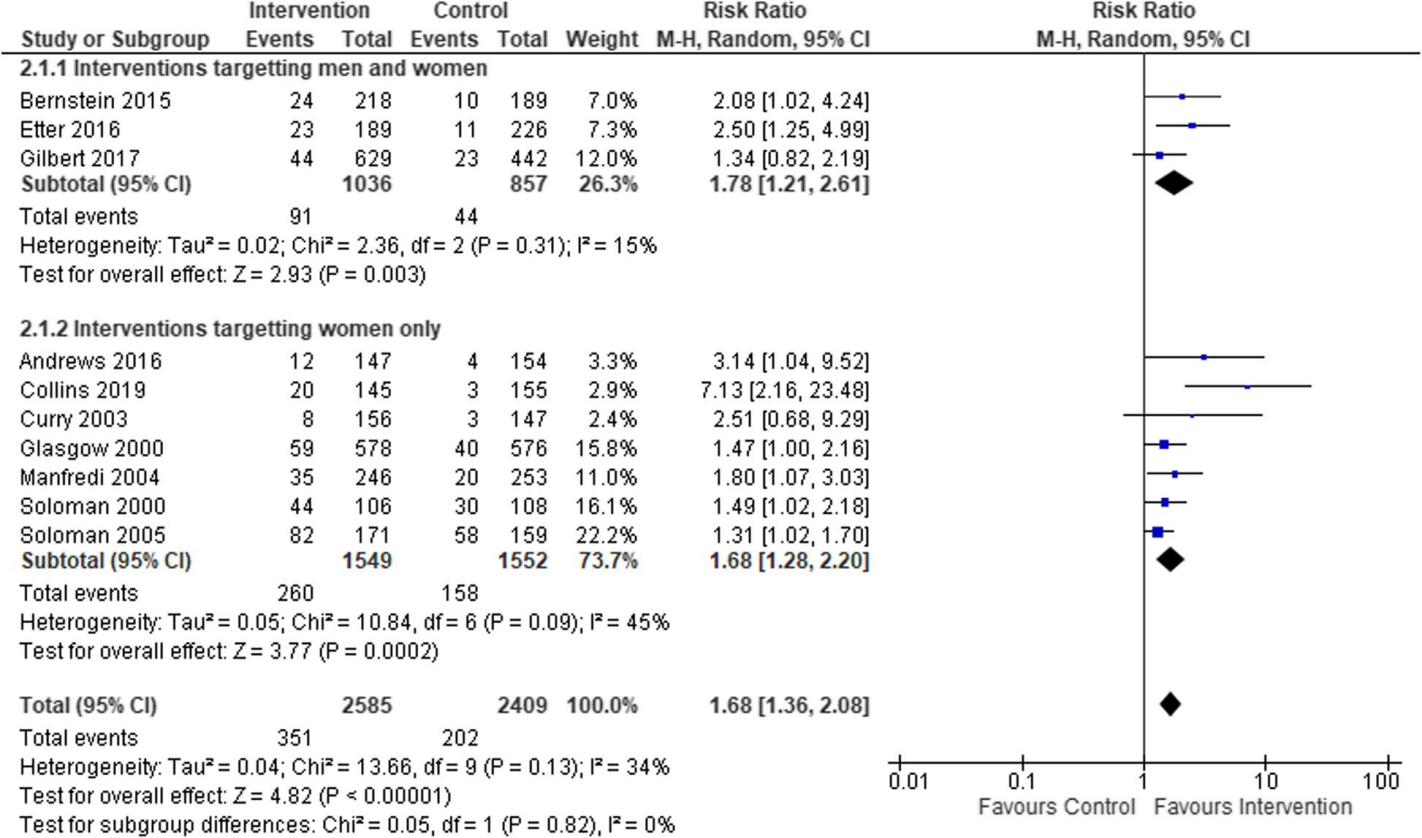
Interventions targeting only women versus interventions targeting both men and women

**Fig. 6:**
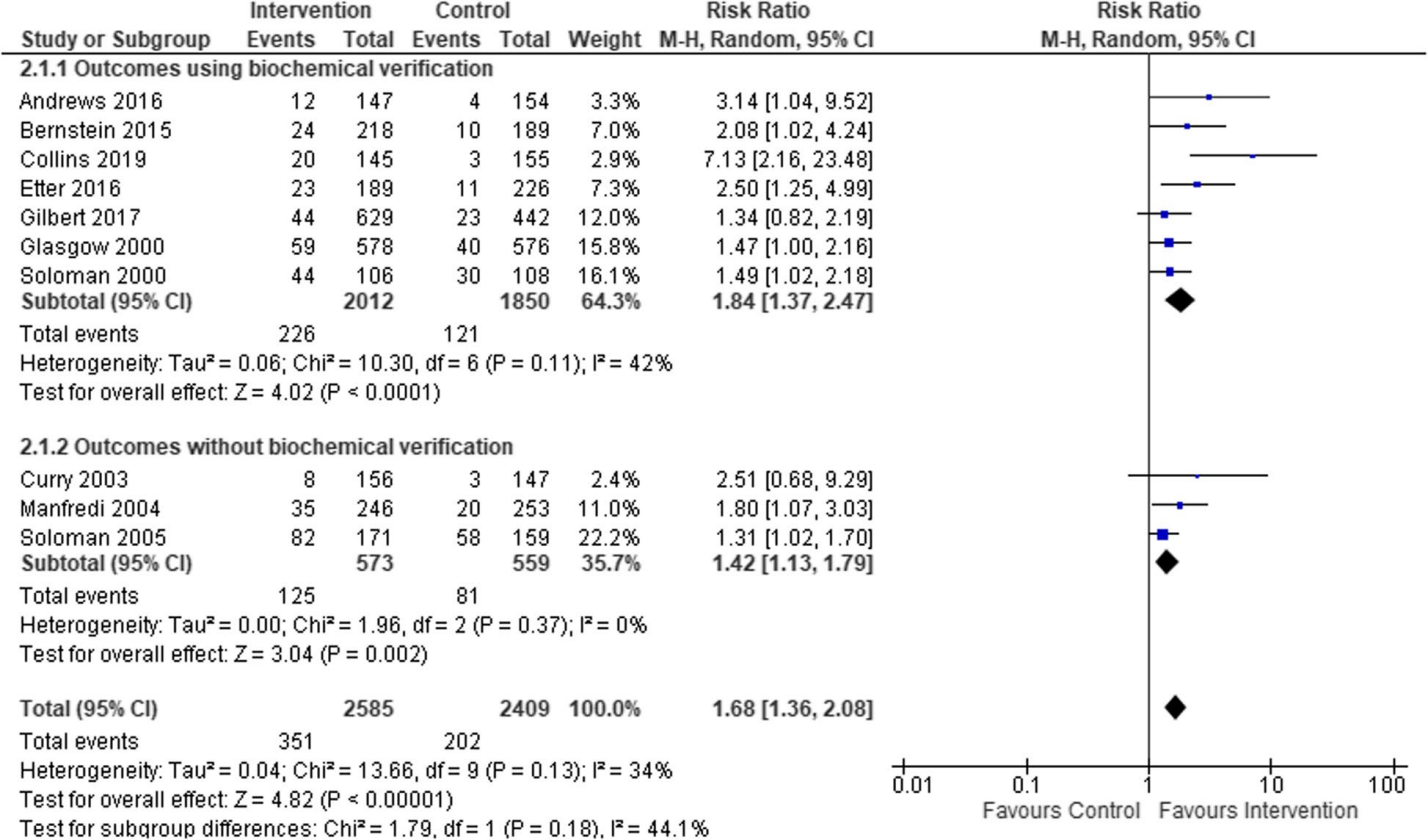
Comparison of interventions which included biological verification of smoking cessation versus interventions that did not

**Fig. 7:**
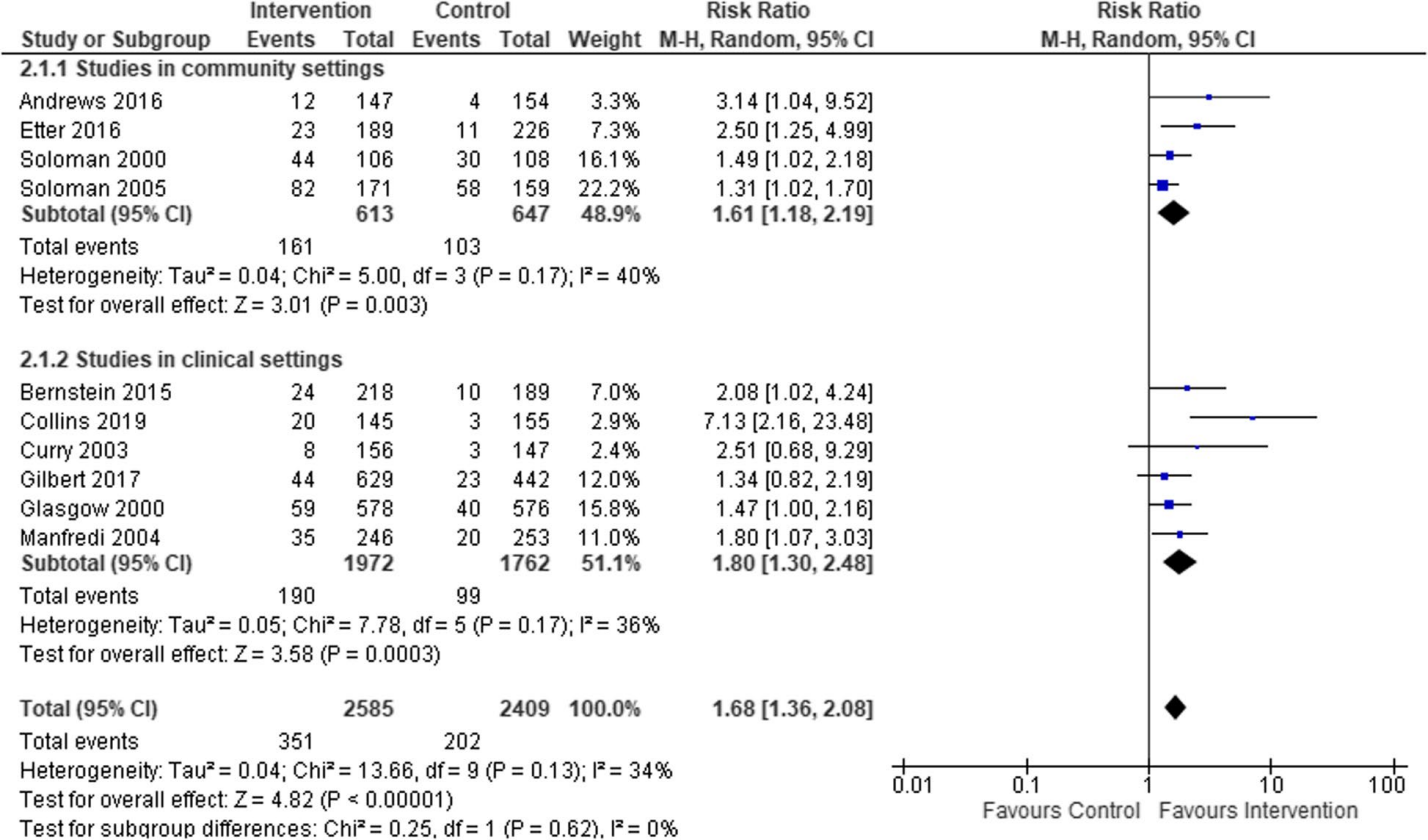
Comparison of studies taking place in clinical settings versus studies in communities

**Fig. 8:**
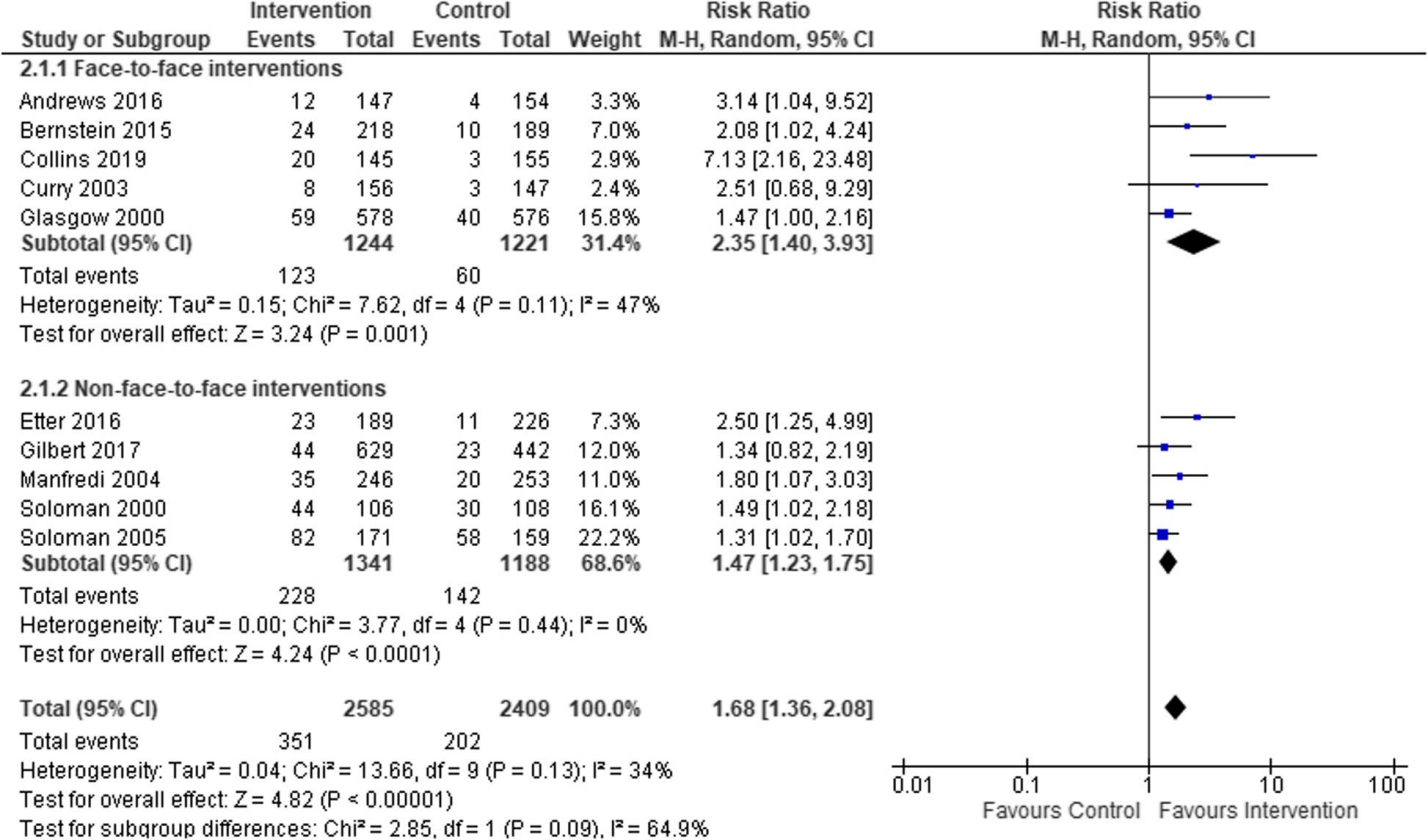
Comparison of face-to-face interventions versus other intervention types

**Fig. 9:**
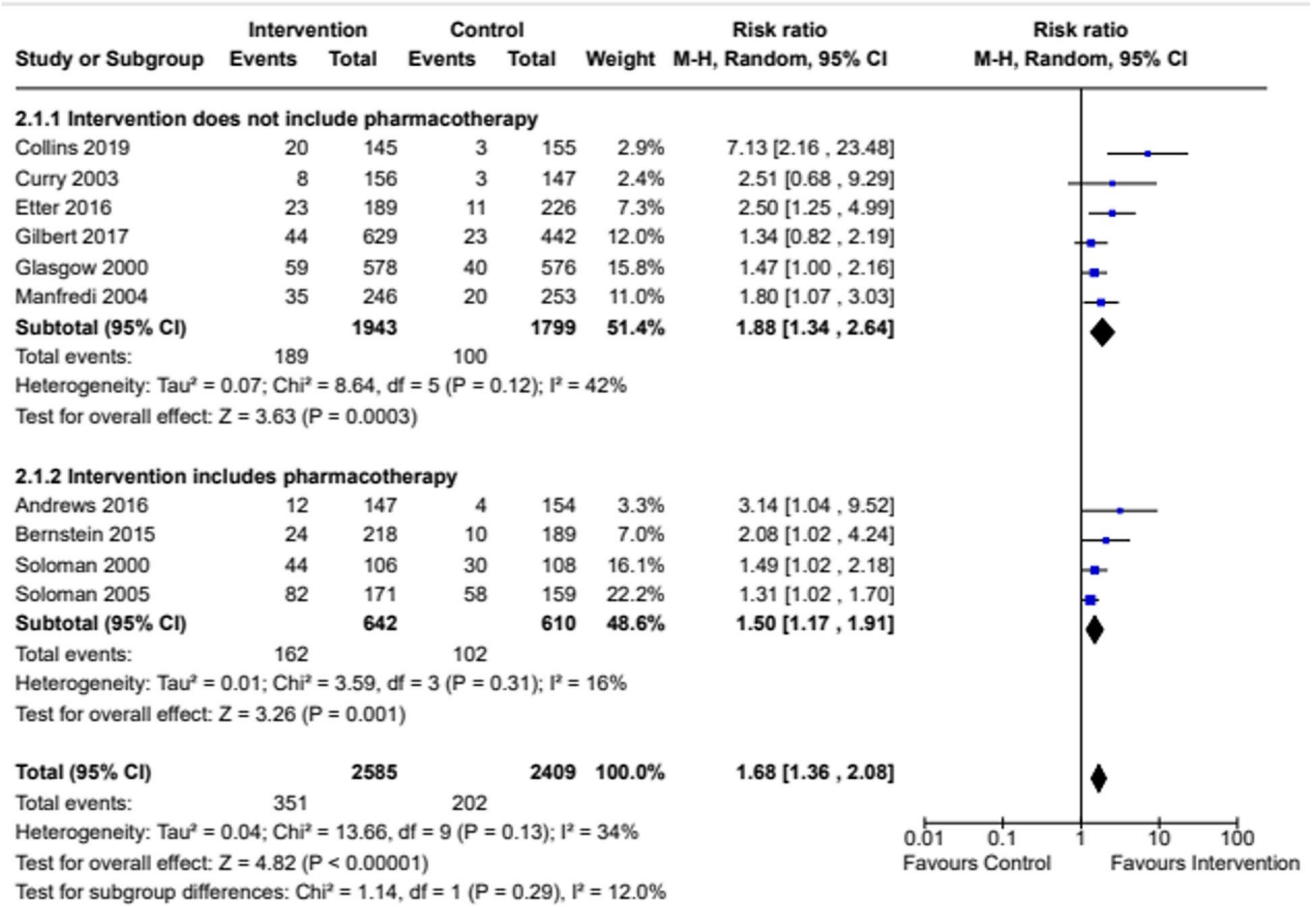
Comparison of studies testing intervention which included pharmacological therapies versus those that did not include pharmacological therapies

We compared smoking cessation outcomes in low-SES women, who had attended interventions targeting women-only, to low-SES women attending interventions targeting both men and women. Both intervention types found a statistically significant effect in favour of interventions compared to controls (see Fig. 5), but no statistical difference between subgroups. Studies that included biochemical verification and those that did not both had statistically significant effects in favour of the intervention, but again no significant difference between subgroups (see Fig. 6). Studies based in clinical settings and those based in communities both had statistically significant effects in favour of the intervention compared to control arms, with no statistical difference between subgroups (Fig. 7). There was a greater effect size in face-to-face interventions compared to other types of interventions, however, both intervention types favoured interventions rather than controls (see Fig. 8). Again, there was no statistically significant difference between subgroups (*p* = 0.09). Pharmacological therapies were included in four of the interventions and were not included in six. Although effectiveness was noted in interventions with pharmacotherapy and without, there was no significant difference in effectiveness between subgroups (Fig. 9).

## Discussion

The findings of this systematic review and meta-analysis demonstrate that smoking cessation interventions directed to low-SES women are effective in the short term and that the effect persists but decreases with longer follow-up.

Previous reviews of smoking cessation interventions show promise in improving smoking cessation rates in disadvantaged groups including at-risk youth, individuals with mental illness and low-income smokers [32, 33]. Bryant et al. [32] conducted a meta-analysis of effectiveness of smoking cessation interventions for low-income females using three trials (*n* = 2525) and found a significant effect for studies when measured at the shortest follow-up interval, though not when measured at the longest available interval. All three studies however originated in prenatal or paediatric clinics and, as such, have little generalisability for women who do not attend such services. Kock et al. [33] examined the effectiveness of interventions for disadvantaged groups and the degree to which effectiveness is moderated by the tailoring of SES. They found evidence that individual-level interventions have some effectiveness for disadvantaged smokers but provide no information on gender-specific interventions.

We identified 11 studies that met our inclusion criteria. These were published between 1997 and 2019 and included a total of 6153 female participants. Studies varied in setting, recruitment methods, fidelity measures, intervention duration and type, comparison conditions and follow-up length.

The difference in smoking cessation rates between interventions and controls in this review at intervention end (RR 1.68, 95% CI 1.36–2.08) is similar to risk ratios reported in previous meta-analyses, for example the pooled cessation rate in individual-level interventions in socioeconomically disadvantaged groups generally (RR 1.56, 95% CIs 1.39–1.75) [33]; the rate in reduction-to-quit interventions compared to no treatment (RR 1.74, 95% CIs 0.90–3.38, although not significant) [57]; and the rate in smoking cessation interventions in hospital settings (pooled odds ratio: 1.65, 95% CI 1.44–1.90) [58]. It is worth noting that while there was a statistically significant difference between rates in the control and intervention groups in our study, the proportion of women stopping smoking was low (ranging from 7 to 47.9% in the intervention group). While absolute success rates are low, when applied to low-SES women in any specific country, these interventions can have a large effect. Smoking cessation may also have a cyclical nature, whereby successful quitting requires several initial quitting attempts, often up to 30 or more before sustained quitting [59].

Few countries have attempted to tailor smoking cessation services to meet the needs of women [60]. Hence, developing effective ways for low-SES women to engage with smoking cessation services is a public health priority. A recent development to address this need is the ‘We Can Quit’ programme developed in local communities in Ireland using a community-based participatory research approach which is currently being evaluated in a pilot RCT [61].

Face-to-face interventions for smoking cessation demonstrated the strongest effect size compared to other intervention types (although it is important to note there was no statistical difference between subgroups). A previous review, which included men and women, found individual face-to-face counselling was more effective than self-help materials, and more intensive forms of counselling were more effective than less intensive interventions [62]. It is noteworthy and a cause for optimism that the effect sizes in the overall meta-analysis favoured the intervention groups even when 8/11 studies included in the meta-analysis used active controls, rather than treatment-as-usual or no treatment. In these studies, active controls included written materials, NRT and Quitline telephone numbers.

Slightly higher effect sizes amongst women were observed in the three studies that targeted both men and women, rather than in the seven studies which targeted women only, although there was no statistical difference between subgroups. It is possible studies that targeting both genders include male and female family members, who can support each other in their attempts to stop smoking. Future research comparing studies tailored specifically to low-SES men and low-SES women, as well as comparisons of smoking cessation intervention effectiveness in low-SES women compared to affluent women, would be informative. Similarly, as this review includes only one study with a female-only group intervention [48], one cannot draw definitive conclusions on the effectiveness of gender-specific individual-level or gender-specific group interventions, or the role of possible mechanisms like group cohesion or camaraderie in gender specific programmes.

Studies that included biochemical verification in their smoking cessation outcome measure had greater pooled effect sizes compared to studies with self-report outcomes only (although the difference between subgroups was not statistically significant potentially due to the small number of studies included in the review), suggesting participants self-reporting smoking may not over-report cessation compared to those giving biochemical samples. Studies taking place in clinical settings also had a marginally higher effect size than community-based studies, although again the subgroups were not statistically significantly different. There may be a higher rapport between clinicians and participants which may be easier to capitalise during an intervention compared to community-based studies which require de novo relationship- and rapport-building. The skill of the clinical practitioner is also likely to play a role, as well as potentially greater precision in data collection, although this is not something we could quantify. It is important to note that while none of our subgroups showed statistically significant differences, this may have been due to the relatively small number of studies included.

Ten of the 11 studies used point-prevalence outcomes, with nine using 7-day point prevalence, one using 30-day point prevalence and one study using a continuous abstinence outcome. There is some debate over the robustness of the point prevalence measure although both point prevalence and continuous abstinence are highly correlated measures and produce similar effect sizes [63]. The relatively low heterogeneity in this study further supports this.

With the exception of Andrews et al. [48], all studies adopted individual-level approaches to smoking cessation rather than neighbourhood or community-level approaches. Bauld et al. [64] reported that while NHS Stop Smoking Services helped reduce health inequalities, their individual-level approach did not address the social circumstances which perpetuate health inequalities. This can be addressed through directing increased investment to communities where smoking prevalence is highest and viewing individual-level interventions as one part of broader tobacco control policies and strategies and income redistribution.

There are several limitations in this study. A small number of protocol deviations were made. Information on the proportion of participants recruited to trials from those eligible was not obtained as it was not routinely reported.

There was also a lack of consistent information on previous quit attempts, baseline and post-intervention dependence (e.g. Fagerstrom scores) and smoking reduction behaviour which would have been useful additional moderating information. This review included only one group intervention so we could not compare the effectiveness of group sessions versus one-to-one interventions. We did not find information on any adverse effects of interventions such as withdrawal symptoms or serious adverse events which would have been of interest when investigating potential mediating effects of the intervention on abstinence, although serious adverse events are unlikely with NRT, which is an over-the-counter drug. The effectiveness of behavioural support is likely dependent on the skill of the person delivering it [65]; however, a lack of information on deliverer skill and experience means we could not account for this in our analyses. We did not include pregnant women in this review as this is the subject of an on-going review [34].

We cannot comment on whether interventions tailored to women in low-SES groups are superior to interventions directed to all SES groups, or to low-SES men or affluent women as this review focuses on low-SES women only. One study in this review targeted all SES groups; however, we included participant data from women living in deprived areas only [46]. Regarding the generalisability of our findings, an important limitation of our study is that most studies were located in the USA, and none in low-income countries. The definitions of deprivation and disadvantage varied between studies and deprivation is itself a relative concept. Deprivation in Switzerland (with a Gini coefficient of 32.3, where 0 indicates perfect equality, and where 0% of the Swiss population live below the international $1.90 poverty line) has a qualitatively different meaning than in the USA (with a Gini coefficient of 41.5 and where 1.2% live below the poverty line) [66]. Additionally, in studies where selection criteria constituted residence in areas of disadvantage rather than individual-level disadvantage, we cannot ascertain whether high-income women took part. As SES definitions differed considerably, a sub-group comparison was not possible.

Follow-up rates varied considerably between studies with three reporting outcomes at the end of treatment only [47, 49, 51]. There are calls for trials to assess abstinence at least 6 months after baseline [57], with preferred follow-ups at 6 and/or 12 months, with or without intermediate measurements [67]. Nine of the included studies report outcomes 6 months post-baseline, but few followed participants beyond this.

Finally, the certainty of the evidence in this review was low. The reason for this was because the risk of bias assessment indicated concerns in most studies, with high risk identified in five of the 11 studies. Imprecision was a concern as the number of events for each outcome was less than 300 in all studies except Gilbert et al. [46], Glasgow et al. [52] and Manfredi et al. [54] (this was further reduced to account for ICC).

While we have downgraded our findings for publication bias, as small negative trials may not have been published, we acknowledge that funnel plots to evaluate publication bias are difficult to interpret, especially with a small number of studies, as data are not adequate to evaluate clear patterns and asymmetry may be due to other factors. These include: industry funded studies, study quality, the different intensity of intervention, differences in underlying risk, choice of effect measure and chance [68].

We therefore must conclude, that the certainty of available evidence on smoking cessation in low-SES is relatively poor.

Despite these limitations, this review has several strengths. With the exception of Bryant et al.’s small meta-analysis, no review had previously examined the effectiveness of smoking cessation interventions among low-SES women or women living in socioeconomically disadvantaged areas. Given the specific adverse effects of smoking in women, the high and sustained rates of smoking in low-SES women, the rapid rise in lung cancer rates in women, coupled with the WHO’s call for smoking cessation interventions tailored to women, our study goes some way in better understanding how low-SES women respond to smoking cessation interventions. This review more generally addresses a lack of information on equity-related issues in tobacco cessation literature. A recent scoping review found only 24.5% of tobacco control reviews addressed equity-related concepts, and the majority of these sought to identify and describe disparities, rather than to account for inequity in relation to intervention effectiveness [69]. This study also benefits from the inclusion of subgroup comparisons allowing the assessment of the size of treatment effects in methodologically different studies.

Future systematic reviews would benefit from reporting the most effective intervention components using a theory-informed taxonomy [22] and consistent trial reporting on intervention attendance, fidelity measures and training sessions for delivery staff would assist in the pooling of data and strengthen future reviews. There is a need for untargeted smoking cessation interventions to publish or make disaggregated data on gender, level of education, SES and ethnicity available for future researchers. Future reviews assessing the cost-effectiveness of smoking cessation interventions for women in low-SES groups and reviews comparing the effectiveness of interventions tailored specifically to low-SES women to those tailored specifically to low-SES men may help shape the design and implementation of tobacco cessation services for those who need them most.

## Conclusions

This is the first systematic review to examine the effectiveness of smoking cessation interventions in socioeconomically disadvantaged women or women living in areas of socioeconomic disadvantage, a group historically under-served by smoking cessation services. Our findings show that behavioural and behavioural plus pharmacotherapy interventions for smoking cessation targeting women in low-SES groups were effective in the short term, though longer follow-up periods indicated reduced effectiveness. Unfortunately, the certainty of available evidence is low. Future studies should explore ways to prevent smoking relapse in low-SES women who have successfully quit smoking and the cost effectiveness of these studies.

## Supplementary Information

**Additional file 1.** Search strategy.

**Additional file 2.** PRISMA checklist.

**Additional file 3.** Accounting for the design effect in cluster RCTs.

## Data Availability

The datasets used and analysed during the current study are available from the corresponding author on reasonable request.

## Abbreviations

BCT: Behavioural change techniques
CI: Confidence interval
GRADE: Grades of Recommendation Assessment, Development and Evaluation
ICC: Intra-class correlation
ITT: Intention to treat
M-H Method: Mantel-Haenszel method
NHS: National Health Service
NRT: Nicotine replacement therapy
PRISMA: Preferred Reporting Items for Systematic Reviews and Meta-analyses
RCT: Randomised controlled trial
RR: Risk ratio
SES: Socio-economic status
TIDieR: Template for intervention description and replication
WHO: World Health Organisation
