# Supplementary material for: The effectiveness of smoking cessation interventions for socio-economically disadvantaged women: a systematic review and meta-analysis
Source: Syst Rev. 2022 Jun 2;11:111. doi: 10.1186/s13643-022-01922-7 (PMC9164420; doi:10.1186/s13643-022-01922-7)
Supplement: Supplementary file 1 — Additional file 1. Search strategy. [file 13643_2022_1922_MOESM1_ESM.docx]

Supplementary Material 1: Systematic review search strategy for each database

| **Database** | **Full search string** | **Number of returned studies** |
| --- | --- | --- |
| **Medline (OVID)** | 1. (exp WOMEN/ or Female/) and (exp POVERTY/ or "Socioeconomic Factors"/ or UNEMPLOYMENT/) 2. ((Wom?n OR female* OR girl* OR mother*) adj5 (disadvantage* OR low* income* OR Low* Socioeconomic OR poverty OR deprivation OR deprive* OR inequalit* OR unemploy*)).ti,ab. 3. or/1-2 4. smoking cessation.mp. or exp Smoking Cessation/ 5. "Tobacco-Use-Cessation"/ 6. "TOBACCO USE CESSATION PRODUCTS"/ 7. "Tobacco-Use-Disorder"/ 8. ((quit$ or stop$ or ceas$ or giv$ or replace* or Abstinence or abstain* or prevent*) adj5 (smoking or tobacco or nicotine or cigarette*)).ti,ab. 9. exp Smoking/pc, th 10. or/4-9 11. exp "Community Health Services"/ or "Community Health Planning"/ or "Community Medicine"/ or "Community Health Centers"/ or "Community Health Workers"/ 12. ((Communit* or group*) adj4 (intervention* OR programme* OR program* OR setting* OR group* OR engage* OR care)).ti,ab. 13. or/11-12 14. and/3,10,13 15. Randomized controlled trials as Topic/ 16. Randomized controlled trial/ 17. Random allocation/ 18. Double blind method/ 19. Single blind method/ 20. Clinical trial/ 21. Exp Clinical Trials as Topic/ 22. or/15-21 23. (clinic$ adj trial$1).tw. 24. ((singl$ or doubl$ or treb$ or tripl$) adj (blind$3 or mask$3)).tw. 25. Placebos/ 26. Placebo$.tw. 27. Randomly allocated.tw. 28. (allocated adj2 random).tw. 29. or/23-28 30. 22 or 29 31. Case report.tw. 32. Letter/ 33. Historical article/ 34. Review of reported cases.pt. 35. Review, multicase.pt. 36. or/31-35 37. 30 not 36 38. and/14,37 | 150 |
| **EMBASE** | 1. ('poverty'/exp OR 'lowest income group'/exp OR 'unemployment'/exp) AND ('female'/exp OR 'mother'/exp)  2. ((Wom?n OR female* OR girl* OR mother*) NEAR/5 (disadvantage* OR ‘low* income*’ OR ‘Low* Socioeconomic’ OR poverty OR deprivation OR deprive* OR inequalit* OR unemploy*)):ti,ab  3. #1 OR #2  4, 'smoking cessation'/exp OR 'smoking ban'/exp  5. ((quit* or stop* or ceas* or giv* or replace* OR cessation OR Abstinence OR abstain* OR prevent*) NEAR/5 (smoking or tobacco or nicotine OR cigarette*)):ti,ab  6. #4 OR #5  7. 'community care'/exp  8. ((Communit* or group*) NEAR/4 (intervention* OR programme* OR program* OR setting* OR group* OR engage* OR care)):ti,ab  9. #7 OR #8  10. #3 AND #6 AND #9  11. 'crossover procedure':de OR 'double-blind procedure':de OR 'randomized controlled trial':de OR 'single-blind procedure':de OR (random* OR factorial* OR crossover* OR cross NEXT/1 over* OR placebo* OR doubl* NEAR/1 blind* OR singl* NEAR/1 blind* OR assign* OR allocat* OR volunteer*):de,ab,ti | 112 |
| **Cochrane Database** | 1. (([mh “Women”] OR [mh “female”]) AND ([mh “Poverty”] OR [mh "Socioeconomic Factors"] OR [mh “Unemployment”]))  2. ((Wom?n OR female* OR girl* OR mother*) NEAR/5 (disadvantage* OR “low* income*” OR “Low* Socioeconomic” OR poverty OR deprivation OR deprive* OR inequalit* OR unemploy*)):ti,ab,kw  3. #1 OR #2  4.[mh “smoking cessation”] OR [mh "Tobacco-Use-Cessation"] OR [mh "TOBACCO USE CESSATION PRODUCTS"]  5. ((quit* or stop* or ceas* or giv* or replace* OR cessation OR Abstinence OR abstain* OR prevent*) NEAR/5 (smoking or tobacco or nicotine OR cigarette*)):ti,ab,kw  6. #4 OR #5  7. [mh “Community care”]  8. ((Communit* or group*) NEAR/4 (intervention* OR programme* OR program* OR setting* OR group* OR engage* OR care)):ti,ab,kw  9. #7 OR #8  10. #3 AND #6 AND #9 | 35 |
| **CINAHL** | 1. (([mh “Women”] OR [mh “female”]) AND ([mh “Poverty”] OR [mh "Socioeconomic Factors"] OR [mh “Unemployment”]))  2. ((Wom?n OR female* OR girl* OR mother*) NEAR/5 (disadvantage* OR “low* income*” OR “Low* Socioeconomic” OR poverty OR deprivation OR deprive* OR inequalit* OR unemploy*)):ti,ab,kw  3. #1 OR #2  4. [mh “smoking cessation”] OR [mh "Tobacco-Use-Cessation"] OR [mh "TOBACCO USE CESSATION PRODUCTS"]  5. ((quit* or stop* or ceas* or giv* or replace* OR cessation OR Abstinence OR abstain* OR prevent*) NEAR/5 (smoking or tobacco or nicotine OR cigarette*)):ti,ab,kw  6. #4 OR #5  7. [mh “Community care”]  8. ((Communit* or group*) NEAR/4 (intervention* OR programme* OR program* OR setting* OR group* OR engage* OR care)):ti,ab,kw  9. #7 OR #8  10. #3 AND #6 AND #9  11. ( (MH "Random Assignment") or (MH "Random Sample+") or (MH "Crossover Design") or (MH "Clinical Trials+") or (MH "Comparative Studies") or (MH "Control (Research)+") or (MH "Control Group") or (MH "Factorial Design") or (MH "Quasi-Experimental Studies+") or (MH "Placebos") or (MH "Meta Analysis") or (MH "Sample Size") or (MH "Research, Nursing") or (MH "Research Question") or (MH "Research Methodology+") or (MH "Evaluation Research+") or (MH "Concurrent Prospective Studies") or (MH "Prospective Studies") or (MH "Nursing Practice, Research-Based") or (MH "Solomon Four-Group Design") or (MH "One-Shot Case Study") or (MH "Pretest-Posttest Design+") or (MH "Static Group Comparison") or (MH "Study Design") or (MH "Clinical Research+") ) or ( clinical nursing research or random* or cross?over or placebo* or control* or factorial or sham* or meta?analy* or systematic review* or blind* or mask* or trial*)  12. S10 AND S11 | 122 |
| **PsychINFO** | 1. (DE "Human Females" OR DE "Mothers" OR DE "Sisters" OR DE "Widows" OR DE "Wives" OR "Unwed Mothers" ) AND (DE "Poverty" OR DE "Disadvantaged" OR DE "Homeless" OR DE "Income (Economic)" OR DE "Lower Income Level" OR DE "Socioeconomic Status" OR DE "Unemployment")  2. TI ((Wom?n OR female* OR girl* OR mother*) N5 (disadvantage* OR “low* income*” OR “Low* Socioeconomic” OR poverty OR deprivation OR deprive* OR inequalit* OR unemploy*)) OR AB ((Wom?n OR female* OR girl* OR mother*) N5 (disadvantage* OR “low* income*” OR “Low* Socioeconomic” OR poverty OR deprivation OR deprive* OR inequalit* OR unemploy*))  3. S1 OR S2  4. DE "Smoking Cessation"  5. TI ((quit* or stop* or ceas* or giv* or replace* OR cessation OR Abstinence OR abstain* OR prevent*) N5 (smoking or tobacco or nicotine OR cigarette*)) OR AB ((quit* or stop* or ceas* or giv* or replace* OR cessation OR Abstinence OR abstain* OR prevent*) N5 (smoking or tobacco or nicotine OR cigarette*))  6. S4 OR S5  7. (DE "Community Advocacy" OR DE "Community Counseling" OR DE "Community Health" OR DE "Community Involvement" OR DE "Community Services" OR DE "Community Welfare Services")  8. TI ((Communit* or group*) N4 (intervention* OR programme* OR program* OR setting* OR group* OR engage* OR care)) OR AB ((Communit* or group*) N4 (intervention* OR programme* OR program* OR setting* OR group* OR engage* OR care))  9. S7 OR S8  10. S3 AND S6 AND S9  11. SU.EXACT("Treatment Effectiveness Evaluation") OR SU.EXACT.EXPLODE("Treatment Outcomes") OR SU.EXACT("Placebo") OR SU.EXACT("Followup Studies") OR placebo* OR random* OR "comparative stud*" OR clinical NEAR/3 trial* OR research NEAR/3 design OR evaluat* NEAR/3 stud* OR prospectiv* NEAR/3 stud* OR (singl* OR doubl* OR trebl* OR tripl*) NEAR/3 (blind* OR mask*)  12. S10 AND S11 | 6 |
| **Web of Science** | TS=(((Wom?n OR female* OR girl* OR mother*) NEAR/5 (disadvantage* OR “low* income*” OR “Low* Socioeconomic” OR poverty OR deprivation OR deprive* OR inequalit* OR unemploy*)) AND ((quit* or stop* or ceas* or giv* or replace* OR cessation OR Abstinence OR abstain* OR prevent*) NEAR/5 (smoking or tobacco or nicotine OR cigarette*)) AND ((Communit* or group*) NEAR/4 (intervention* OR programme* OR program* OR setting* OR group* OR engage* OR care))) AND (TS= clinical trial* OR TS=research design OR TS=comparative stud* OR TS=evaluation stud* OR TS=controlled trial* OR TS=follow-up stud* OR TS=prospective stud* OR TS=random* OR TS=placebo* OR TS=(single blind*) OR TS=(double blind*)) | 113 |
| Total number of articles returned: 538; Duplicates: 232; Total unique articles: 398 | | |
