# Supplementary material for: The effectiveness of smoking cessation interventions for socio-economically disadvantaged women: a systematic review and meta-analysis
Source: Syst Rev. 2022 Jun 2;11:111. doi: 10.1186/s13643-022-01922-7 (PMC9164420; doi:10.1186/s13643-022-01922-7)
Supplement: Supplementary file 3 — Additional file 3. Accounting for the design effect in cluster RCTs. [file 13643_2022_1922_MOESM3_ESM.docx]

# Supplementary Materials 3: Accounting for the design effect in cluster RCTs

With an average cluster size of 29, the design effect for Andrew et al.’s study (39) was calculated as 1.36. For the purposes of our meta-analysis, the intervention sample is recalculated as 147, rather than 200 and the control group sample is 154 instead of 209. The number of women who successfully quit smoking in the intervention group is 12, rather than 16 and in the control group 4, rather than 6. With an average cluster size of 89, the design effect for Manfredi et al.’s study (40) is 2.14 giving an adjusted intervention sample size of 246 rather than 527 and a control group sample of 253 rather than 541. The number of women who successfully quit is therefore 35, rather than 75 and 20, rather than 42.
